# An Exploratory Study of *ADIPOQ* Polymorphisms, Adiponectin Levels and Metabolic Syndrome in a Vietnamese Population

**DOI:** 10.3390/ijms27041780

**Published:** 2026-02-12

**Authors:** Phat Tung Ma, Nam Quang Tran, Minh Phuc Ha Vu, Le Gia Hoang Linh, Nien Vinh Lam

**Affiliations:** 1 Department of Endocrinology, University Medical Center, Ho Chi Minh City 700000, Vietnam; phat.mt@umc.edu.vn; 2 Department of Endocrinology, School of Medicine, University of Medicine and Pharmacy, Ho Chi Minh City 700000, Vietnam; 3 Faculty of Public Health, University of Medicine and Pharmacy, Ho Chi Minh City 700000, Vietnam; 4 Center of Molecular Biomedicine, University of Medicine and Pharmacy, Ho Chi Minh City 700000, Vietnam; 5 Department of Biochemistry, School of Medicine, University of Medicine and Pharmacy, Ho Chi Minh City 700000, Vietnam

**Keywords:** *ADIPOQ*, adiponectin, metabolic syndrome, Vietnamese

## Abstract

Metabolic syndrome (MtS) is a growing global health concern, with genetic factors playing a significant role in its development. This study aimed to examine the association between *ADIPOQ* polymorphisms and MtS, as well as their relationships with adiponectin levels in the Vietnamese population. Metabolic parameters and genotyping data were collected from 160 individuals diagnosed with MtS according to the 2005 International Diabetes Federation criteria, and 160 control subjects. The results reveal a significant association between rs266729 and MtS, with a log-additive OR = 1.46 (95% CI: 1.02–2.09, *p* = 0.038). Carriers of the G allele also exhibit lower median adiponectin levels: 6.69 (3.84–10.52) µg/mL for C/C, 6.55 (3.32–9.52) µg/mL for G/C, and 4.28 (3.14–6.38) µg/mL for G/G, *p* for trend = 0.036. In contrast, the associations of rs2241766 and rs1501299 with MtS did not reach statistical significance in this cohort. However, adiponectin levels also show a trend of progressively decreasing with increasing numbers of minor alleles in the genotypes, particularly in the control group, *p* for trend = 0.02 and 0.044, respectively. These findings underscore the importance of genetic variations in regulating adiponectin, especially during the early stages of metabolic disturbances, and call for further research to assess their clinical significance.

## 1. Introduction

Metabolic syndrome (MtS) refers to a group of interlinked metabolic abnormalities—central obesity, elevated fasting glucose, increased blood pressure (BP), and dyslipidemia—that together double the risk of cardiovascular disease and increase the risk of developing diabetes fivefold [1]. MtS is a common condition and a frequent focus of epidemiological research in Vietnam because of its substantial health impact [2,3]. In a meta-analysis comprising 18 studies with a total of 35,421 participants, the pooled prevalence of MtS among Vietnamese adults was estimated at 16.1%; greater prevalence was observed in females, urban residents, individuals with elevated body mass index (BMI), and those with a higher body fat percentage [4]. Given these patterns and the broad cardiometabolic consequences of MtS, it is increasingly recognized as a key target for early prevention in contemporary healthcare.

Unhealthy lifestyle factors, including physical inactivity [5], unhealthy diet [6], and excessive alcohol consumption [7], play a significant role in the progression of MtS. In addition, genetic predisposition contributes to the syndrome’s pathogenesis, with individuals having a family history of MtS exhibiting a markedly higher risk; several gene variants are implicated in obesity-related mechanisms [8,9].

Adiponectin, a protein hormone mainly secreted by fat tissue, plays a key role in regulating glucose and lipid metabolism, with anti-inflammatory and anti-atherogenic effects. Reduced adiponectin levels are commonly observed in individuals with metabolic disorders, such as obesity [10,11] and diabetes [12,13]. Given this link, genetic variations in the *ADIPOQ* gene, which encodes adiponectin, have become a major focus in efforts to elucidate the pathogenesis of MtS.

Among these genetic variations, several single-nucleotide polymorphisms (SNPs), such as rs266729, rs2241766, and rs1501299, have been investigated for their potential impact on adiponectin expression and susceptibility to MtS. However, findings across studies remain inconsistent and at times contradictory. Some studies report strong associations between specific SNPs and reduced adiponectin levels [11,14,15,16,17] or increased risk of MtS [18,19,20,21], whereas others fail to replicate these results in different populations or under similar study conditions [22,23,24,25].

These discrepancies highlight the complexity of the *ADIPOQ*–adiponectin–MtS axis. A previous study in a Vietnamese cohort identified associations between *ADIPOQ* variants and type 2 diabetes, with only incidental examination of MtS. Notably, MtS was not a prespecified outcome, and central obesity was assessed using anthropometric thresholds not tailored to Asian populations [26]. Furthermore, the potential link between these polymorphisms and circulating adiponectin levels remains unexplored, leaving an important gap in understanding the continuum from genetic variation to adiponectin dysregulation and downstream metabolic disturbances. To address this gap, the present study was designed to investigate the relationships between *ADIPOQ* polymorphisms, circulating adiponectin concentrations, and MtS in the Vietnamese population.

## 2. Results

### 2.1. Patient Characteristics

Between September 2023 and March 2025, 320 participants were consecutively enrolled in this case–control study conducted at the University Medical Center, a tertiary hospital in the south of Vietnam. By design, 160 patients with MtS and 160 controls were matched by age and sex, which resulted in comparable demographic profiles between the groups. The mean age was similar (47.33 ± 9.07 years in MtS vs. 47.61 ± 8.88 years in the control group; *p* = 0.784), and the sex ratio was 1:1 in both groups.

In contrast, MtS patients exhibited significantly higher values for key anthropometric characteristics. Both BMI and waist circumference (WC) were markedly higher (26.57 ± 3.0 vs. 21.32 ± 1.94 kg/m^2^ and 92.91 ± 8.32 vs. 76.83 ± 5.66 cm for the MtS and control groups, respectively, both *p* < 0.001). The waist-to-hip (WHR) ratio was also elevated in the MtS group (0.94 ± 0.07 vs. 0.86 ± 0.05; *p* < 0.001), and a substantially greater proportion of MtS patients had increased WHR (91.88% vs. 32.5%; *p* < 0.001).

Regarding metabolic bioparameters, the MtS group demonstrated more pronounced abnormalities (Table 1). High-density lipoprotein (HDL) cholesterol levels were significantly lower (1.14 ± 0.25 vs. 1.38 ± 0.3 mmol/L; *p* < 0.001), while systolic and diastolic BP were notably higher (134.19 ± 14.94 vs. 120.94 ± 12.84 mmHg and 85.24 ± 11.35 vs. 77.12 ± 8.78 mmHg, respectively; *p* < 0.001). Mean fasting glucose was elevated in the MtS group (6.72 ± 1.56 vs. 5.09 ± 0.37 mmol/L; *p* < 0.001), along with significantly higher median levels of triglycerides (2.41 [1.86–3.3] vs. 1.18 [0.87–1.59] mmol/L; *p* < 0.001) and fasting insulin (53.94 [34.32–81.6] vs. 22.98 [16.14–35.46] pmol/L; *p* < 0.001). Conversely, median adiponectin levels were significantly lower in MtS patients compared to controls (4.44 [2.39–6.83] vs. 9.01 [6.09–12.45] µg/mL; *p* < 0.001). These findings collectively reflect the typical metabolic profile associated with MtS.

As several metabolic parameters have sex-specific reference ranges, we additionally presented sex-stratified statistics. WC, hip circumference, WHR, and HDL cholesterol showed consistent differences between the MtS and control groups in both women and men (*p* < 0.001).

In the MtS group, all patients (100%) presented with central obesity, as defined by increased WC in line with International Diabetes Federation (IDF) diagnostic criteria [27]. As central obesity is the key criterion for MtS, no subjects with elevated WC were included in the control group. Table 2 provides additional data on the prevalence of individual MtS components in both study groups. Among the other components, elevated blood glucose and elevated BP were the most prevalent, observed in 134 (83.75%) and 130 patients (81.25%), respectively. In contrast, these metabolic abnormalities were much less common in the control group. When analyzing the number of MtS components present per individual, most control subjects (83.1%) had none or only one component, whereas no patients in the MtS group had fewer than three components. Specifically, 81 patients (50.6%) in the MtS group had four components, and 39 patients (24.4%) fulfilled all five diagnostic criteria. These findings further emphasize the clustering of metabolic abnormalities in the MtS group compared to controls (*p* < 0.001).

**Figure 1:**
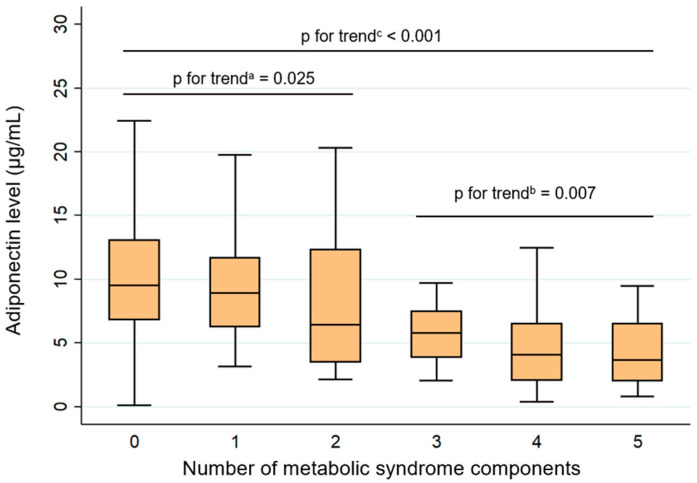
Boxplot showing the distribution of adiponectin levels (µg/mL) according to the number of MtS components. *p* values for assessing trends were determined using Cuzick’s non-parametric trend test, which evaluated changes in adiponectin levels as the number of metabolic syndrome components increased, ^a^ within the control group, ^b^ within the MtS group, and ^c^ in the overall population.

Adiponectin levels decreased gradually with an increasing number of MtS components (Figure 1). Participants without any MtS component had the highest values, whereas those with four or more components showed notably lower concentrations. Specifically, the median and interquartile ranges (IQRs) of adiponectin levels were 9.52 (6.81–13.08), 8.92 (6.25–11.72), 6.42 (3.47–12.34), 5.81 (3.87–7.48), 4.08 (2.06–6.53), and 3.66 (2.03–6.55) µg/mL in subjects with 0, 1, 2, 3, 4, and 5 MtS components, respectively. Trend analyses confirmed a significant inverse association between adiponectin and the accumulation of metabolic abnormalities (*p* for trend < 0.001). In the control group (individuals with two or fewer MtS components), the trend reduction in adiponectin remained significant (*p* = 0.025). A similar pattern was found among individuals with MtS comprising three or more MtS components (*p* = 0.007).

In addition, adiponectin concentrations were higher in females than in males. The median (IQR) values were 7.57 (4.58–11.36) µg/mL in females and 5.35 (2.64–8.35) µg/mL in males (*p* < 0.001). In multivariable linear regression of log-transformed adiponectin (Table S1 in Supplementary Materials), male sex remained associated with lower adiponectin after adjustment for age and MtS status (β = −0.424; 95% CI −0.567 to −0.280; *p* < 0.001).

### 2.2. Genotypic Distribution of ADIPOQ Polymorphisms and Their Association with MtS Risk

Table 3 shows the distribution of *ADIPOQ* genotypes (rs266729, rs2241766, and rs1501299) and their associations with MtS, analyzed under multiple genetic models (dominant, codominant, recessive, and log-additive). Before conducting the association analyses, we evaluated the genotype distributions, allele frequencies, and Hardy–Weinberg equilibrium (HWE). The minor allele frequencies in the total population were 0.23 for rs266729 (G allele), 0.38 for rs2241766 (G allele), and 0.27 for rs1501299 (T allele). The corresponding HWE *p* values in controls were 0.13, 0.41, and 0.41, respectively. The absence of HWE deviation supports the representativeness and genotyping accuracy of the study population for subsequent association analyses.

The G allele of rs266729 presented more frequently among participants with MtS than in controls, suggesting a possible link between this polymorphism and metabolic susceptibility. Under the codominant model, individuals carrying the G allele genotypes exhibited a higher risk of MtS compared with those possessing the C/C genotype (OR = 1.65, 95% CI = 1.05–2.60, *p* = 0.029). A consistent trend was also observed in the log-additive model (OR = 1.46, 95% CI = 1.02–2.09, *p* = 0.038), indicating that each additional copy of the G allele was associated with an increase in MtS risk. For rs2241766, no statistically significant differences in genotype or allele frequencies were observed between the MtS and control groups across all the genetic models tested (*p* > 0.05). Similarly, rs1501299 showed no significant association with MtS under any inheritance model. In the log-additive model, the odds ratio was 1.13 (*p* = 0.49), suggesting the absence of a meaningful effect of the T allele on disease susceptibility.

Sex-stratified genotype distributions and association results are presented in Tables S2 and S3 (Supplementary Materials). rs266729 showed a directionally consistent association with MtS in both females and males, with a stronger signal in males under the log-additive model (OR = 1.54, *p* = 0.084) than in females (OR = 1.38, *p* = 0.23). In contrast, rs2241766 and rs1501299 were not significantly associated with MtS in either sex across the tested models. Additionally, SNP×sex interaction testing under the log-additive model did not provide evidence of effect modification by sex for any of the three variants (*p* for interaction > 0.05; Table S4 in the Supplementary Materials).

**Figure 2:**
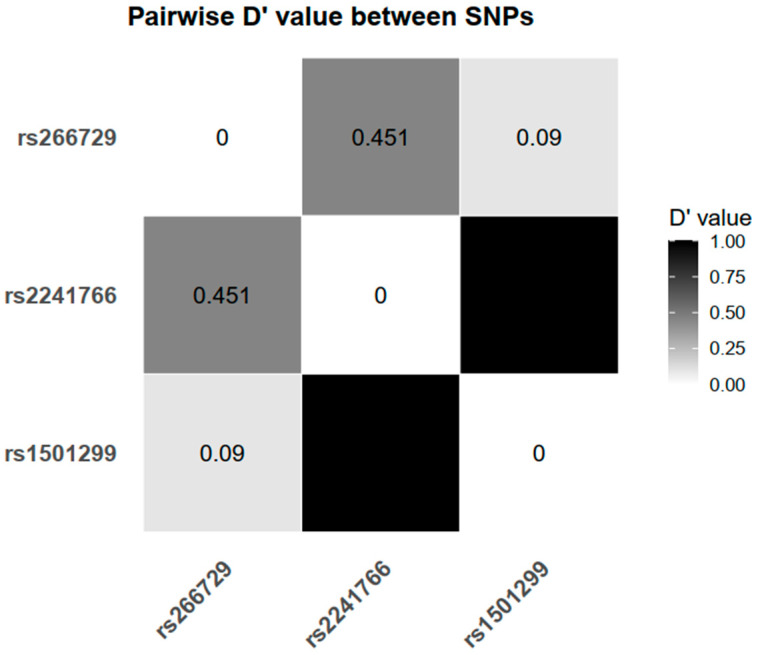
Pairwise linkage disequilibrium among the three *ADIPOQ* SNPs (rs266729, rs2241766, and rs1501299), expressed as D′ values. Each cell shows the D′ value for a SNP pair; grayscale intensity corresponds to D′ (0 = no linkage disequilibrium, 1 = complete linkage disequilibrium), as indicated by the color bar. Abbreviations: SNP, single-nucleotide polymorphism.

Analysis of linkage disequilibrium (LD) among the three *ADIPOQ* polymorphisms revealed that rs2241766 and rs1501299 were in strong LD, with a D′ value of 0.999, indicating that these two loci are likely co-inherited. In contrast, rs266729 showed only moderate LD with rs2241766 (D′ = 0.451) and very weak LD with rs1501299 (D′ = 0.09), suggesting that rs266729 segregates more independently from the other two variants (Figure 2).

### 2.3. Relation of Adiponectin Levels and Genotypes of Each ADIPOQ Gene Variant Between Studied Populations

Table 4 summarizes the variation and trends in serum adiponectin concentrations across the three genotypes of each *ADIPOQ* SNP. The genotypes are displayed in the order of major allele homozygote, heterozygote, and minor allele homozygote. Adiponectin levels were compared among genotypes to assess overall differences, and trend analyses were additionally performed to explore the directional association, assuming that carriers of the minor allele homozygote would have the lowest median adiponectin concentrations.

For rs266729, adiponectin levels showed a significant difference across genotypes in the overall population (*p* = 0.038). The median (IQR) concentrations were 6.69 (3.84–10.52) µg/mL for C/C, 6.55 (3.32–9.52) µg/mL for G/C, and 4.28 (3.14–6.38) µg/mL for G/G genotypes. A gradual decrease in adiponectin concentrations from C/C to G/G was confirmed by the trend analysis (*p* for trend = 0.036). Among controls, adiponectin levels were comparable between C/C and G/C carriers, whereas individuals with the G/G genotype clearly showed lower values (*p* = 0.028). In contrast, the MtS group exhibited neither a significant difference between genotypes nor a discernible trend (*p* for comparison = 0.680; *p* for trend = 0.838).

For rs2241766, no significant trend in adiponectin levels was observed across the three genotypes in the total population (*p* for trend = 0.083). However, a difference in adiponectin concentration was noted, with the lowest median value observed in G/G carriers (*p* for comparison = 0.044). Similarly, among subjects with MtS, adiponectin levels were not different between genotypes under any analysis model. In contrast, within the control group, median (IQR) adiponectin concentrations demonstrated a gradual decrease from 11.84 (8.95–17.21) µg/mL in T/T, to 9.35 (6.88–12.49) µg/mL in G/T, and 7.21 (5.56–10.62) µg/mL in G/G genotypes, forming a consistent downward trend (*p* for trend = 0.020).

For rs1501299, the pattern of adiponectin distribution across genotypes was generally similar to that observed for rs2241766. No significant differences or trends were found in the total population (*p* for comparison = 0.150; *p* for trend = 0.081) or between individuals with MtS (*p* = 0.656). In the control group, however, adiponectin levels showed a slight stepwise decrease, with median (IQR) concentrations of 9.35 (6.58–14.79) µg/mL for G/G, 8.76 (5.94–11.75) µg/mL for G/T, and 5.56 (3.59–9.06) µg/mL for T/T genotypes. Nevertheless, this downward tendency was modest (*p* for trend = 0.044).

## 3. Discussion

The first finding in our study was a progressive decline in adiponectin concentration with increasing metabolic burden. Lower adiponectin levels in individuals with MtS compared with those without the syndrome have been consistently reported in previous literature [24,28,29]. Our findings are in agreement with this pattern. Importantly, adiponectin levels decrease as the number of MtS components increases [12,29]. In a cross-sectional study conducted in Brazil, Frankenberg et al. [12] also reported the lowest adiponectin concentrations among individuals presenting with all five MtS components and demonstrated a negative association between adiponectin levels and the number of MtS abnormalities, similar to our results. However, that study did not provide detailed adiponectin trends for participants without MtS. The reproducibility of this trend in our data indirectly supports the reliability of our MtS component assessment and the validity of our adiponectin measurements. Additionally, our study reveals that this decreasing trend is already apparent among individuals who have only one or two MtS components, i.e., those who have not yet met the criteria for MtS. This result suggests that adiponectin may serve as a sensitive biomarker that reflects early metabolic disturbance and accumulates progressively as cardiometabolic risk increases. Further, Baden et al. [30], using data from a large Japanese cohort, reported that even borderline abnormalities in MtS components, such as high normal BP or impaired fasting glucose, are already associated with reduced adiponectin concentrations. Taken together, these observations reinforce the potential prognostic value of adiponectin in cardiometabolic disease and highlight its relevance for early risk stratification and future predictive or diagnostic modeling.

From a pathophysiological perspective, adiponectin levels are closely linked to each component of MtS. As an adipokine primarily secreted by adipose tissue, adiponectin is strongly associated with total body fat mass, particularly visceral adiposity. Increases in body weight and WC reflect expansion of visceral fat, which adversely affects adipokine secretion, including that of adiponectin. This reduction is mediated through several mechanisms, such as tissue hypoxia, oxidative stress, chronic low-grade inflammation, and mitochondrial dysfunction [31,32]. In addition, adiponectin is closely involved in glucose metabolism and plays a key role in enhancing insulin sensitivity. This effect was proven to be independent of visceral fat mass by Yamamoto [13]. The underlying mechanism is thought to involve multiple pathways, including the inhibition of glucose-6-phosphatase and phosphoenolpyruvate carboxykinase, thereby reducing hepatic gluconeogenesis [33]. Additionally, adiponectin enhances glucose utilization by promoting the translocation of GLUT–4 transporters to the cell membrane [34,35]. In terms of dyslipidemia, adiponectin is positively associated with HDL cholesterol and inversely associated with triglycerides. Adiponectin increases HDL cholesterol by promoting cholesterol efflux and HDL particle formation, and by reducing hepatic lipase activity; these effects have been shown to be independent of BMI and insulin resistance [36]. With respect to triglyceride metabolism, adiponectin promotes the removal of triglyceride-rich lipoproteins, such as chylomicron and very-low-density lipoprotein, by upregulating both the expression and functional activity of lipoprotein lipase [37]. As one of the components of MtS, increased BP is also negatively associated with adiponectin levels [38], and this association is particularly evident in individuals with obesity [39]. The underlying mechanism may relate to the vasculoprotective effects of adiponectin, including its role in enhancing nitric oxide production, reducing sympathetic nervous system activity, and improving endothelial function [38].

Our data demonstrates a modest association between the rs266729 polymorphism and MtS, with an OR of 1.65 under the dominant genetic model. This finding reflects the potential role of the G allele in metabolic dysregulation associated with MtS. However, the associations between rs266729 and metabolic traits have been documented in several studies with conflicting reports [18,19,20,23,24,40,41]. In a Mexican population, this SNP was found to be linked to an increased risk of MtS, with ORs ranging from 2.16 to 4.0 depending on the genetic model analyzed [20]. However, a study involving Hispanic individuals did not observe such an association [24]. Among European populations, Divella et al. also reported a significant relationship between rs266729 and individuals with MtS [18]. In Asia, a study conducted in Sudan found that the G allele confers a 2.15-fold higher risk of MtS compared with the C allele [41]. Likewise, research in Thailand confirmed that carriers of the G allele have greater susceptibility to MtS [19]. Conversely, studies in Chinese populations failed to replicate this association and even suggested a potential protective role of the G allele [23,40]. Truong et al. [26] previously identified an association between rs266729 and type 2 diabetes—a metabolic disorder closely linked to MtS—in a different Vietnamese cohort. They also reported a secondary association with MtS in a subgroup analysis. However, this finding should be cautiously interpreted, as MtS was not a predefined outcome and central obesity was assessed using non-Asian-specific waist circumference cut-offs (≥89 cm for females and ≥101 cm for males). In contrast, our study predefined MtS using the IDF 2005 criteria with Asian-specific thresholds, and applied age- and sex-matching between cases and controls. Overall, our findings align with the previous study in suggesting rs266729 as a potential susceptibility marker in the Vietnamese population, although variations in phenotype definition and study design may partly account for differences in effect estimates.

The next variant examined in this study was rs2241766, an exonic SNP that may influence adiponectin metabolism and contribute to MtS susceptibility. However, we did not observe any association with this polymorphism in our dataset. Previous studies have yielded inconsistent findings. Investigations in Thailand [19], China [23,25], and Venezuela [42] have reported no significant association. In contrast, Zhou et al. [22], through a meta-analysis of Chinese cohorts, identified a significant link between rs2241766 and MtS. Additionally, in a small study among the Temiar population in Malaysia, Zahary et al. [21] reported that the T allele substantially increases MtS risk, with odds ratios ranging from 26.5 to 87.2. Overall, these discrepancies suggest that the impact of rs2241766 may vary considerably across ethnicities. However, given the modest sample size and the typically small effect sizes of common variants, the possibility of a type II error cannot be excluded. This is consistent with the genotype-adiponectin pattern observed in Table 4, which indicated an allele-dependent trend. Taken together, our data do not provide strong evidence for a major effect of rs2241766 on MtS susceptibility in this cohort; nevertheless, modest effects cannot be ruled out and should be evaluated in larger, independent populations.

The third polymorphism analyzed in this study was an intronic SNP, rs1501299. This variant has been reported to be associated with MtS in Hispanic populations [24] and in Sudanese individuals [41]. Among Indian cohorts, Kaur et al. [10] confirmed its association with both MtS and obesity, while Gupta et al. [43] demonstrated a relationship in women with MtS. In contrast, our results did not identify any significant association between rs1501299 and MtS. Likewise, studies conducted in several Chinese populations have also failed to show a clear relationship [23,25]. Gao et al. [23] found only a weak association that disappeared after adjustment for age and sex, while Leu et al. [25] reported a correlation limited to MtS patients with concomitant hypertension, and not among those without hypertension. The absence of a clear association in our study and several East Asian cohorts suggests that rs1501299 may exert population-specific effects or interact with other genetic and environmental factors.

One of the earliest explanations for the association between *ADIPOQ* SNPs and MtS is that these variants may alter adiponectin production, thereby modifying metabolic risk. For this reason, many studies have assessed adiponectin levels alongside metabolic traits [11,14,17,44,45,46,47]. Nonetheless, the available data are inconsistent, and the impact of *ADIPOQ* polymorphisms on disease susceptibility and circulating adiponectin levels may be dissociated. Several studies have reported genetic associations without accompanying differences in adiponectin concentrations [48,49], whereas others have described genetically determined hypoadiponectinemia, even in the absence of a clear increase in susceptibility to metabolic traits [44,50]. For instance, Farooq et al. found that rs1501299 is linked to a higher risk of both type 2 diabetes and MtS; however, adiponectin levels did not differ by genotype in either patients or controls [48]. In contrast, Khan et al., in another study involving Indian individuals, observed no association between rs266729 and diabetes risk compared with controls. Nevertheless, they observed that diabetic patients carrying the G allele exhibit lower adiponectin levels than those without the G allele [44]. Because genetic effects may vary between ethnic groups and be modified by environmental factors, these heterogeneous findings prompted us to undertake a more in-depth evaluation of the associations in our population.

Given that rs266729 has been associated with MtS under several genetic models, we extended the analysis to adiponectin levels, comparing concentrations across genotypes and assessing dose-response trends with increasing numbers of G alleles. As anticipated, rs266729 shows both differences in median adiponectin levels and a stepwise decline with the accumulation of G alleles. Notably, however, this genotype-adiponectin gradient was most apparent in the control group and largely diminished among MtS patients. This pattern suggests that the G allele may primarily influence baseline adiponectin concentrations and metabolic susceptibility during the pre-MtS stage. Once MtS is established, the genetic effect of rs266729 is likely obscured by stronger metabolic and environmental factors, such as visceral adiposity, insulin resistance, and chronic inflammation.

Although rs2241766 and rs1501299 are not associated with either MtS risk or adiponectin concentrations in the whole study population, both variants still demonstrated a modest, allele-dependent downward trend in adiponectin levels. These two SNPs show limited LD with rs266729 in our dataset (Figure 2), suggesting that their effects may be independent. In other words, these variants may function as susceptibility alleles that modulate the threshold for developing MtS, rather than determining adiponectin concentrations in individuals already experiencing significant metabolic disturbances. Taken together, these findings reinforce the concept that genetic influences on adiponectin regulation are more detectable during early, subclinical stages of cardiometabolic dysfunction, before overt MtS becomes fully established.

Sex-related differences may influence fat distribution and, in turn, adiponectin production. In adults, adiponectin levels are generally lower in males than in females [51]. Consistent with this pattern, our data showed higher adiponectin concentrations in females, and this difference remained evident after adjustment for age and MtS status. A plausible mechanistic explanation is that androgens may downregulate adiponectin secretion [52], whereas estrogens may promote more favorable adipose-tissue function [53]. Given these sex-related differences in adipose tissue endocrine activity, sex-stratified evaluation of MtS risk may be clinically and biologically relevant.

To explore whether the genetic contribution to MtS might vary by sex, we extended our analyses using sex-stratified models and formal interaction testing (Tables S2–S4 in the Supplementary Materials). Overall, rs266729 showed directionally consistent associations with MtS in both females and males, with a slightly stronger effect estimate in males; however, interaction testing was not significant. Nevertheless, sex-dependent effects of rs266729 have been reported for related anthropometric traits; Siitonen et al. described a sex–genotype interaction for body weight, with genotype-associated differences reaching statistical significance in women [17]. Because stratification reduces statistical power, subtle sex-specific effects cannot be excluded and should be examined in larger independent cohorts.

This work represents one of the first functional-association studies conducted to investigate MtS in the Vietnamese population. The study examined the continuum from *ADIPOQ* genetic variants to circulating adiponectin levels and, ultimately, to metabolic abnormalities. A strict age- and sex-matching strategy was applied, and MtS was diagnosed using criteria appropriate for Asian populations, thereby improving the validity of the analyses. In addition, the genotyping strategy employed cost-effective laboratory techniques, making the approach feasible for research settings in low-resource countries. Despite these strengths, several limitations should be considered. First, the analysis did not incorporate detailed lifestyle data or include environmental factors, which are known to influence metabolic risk. Second, although *ADIPOQ* contains numerous polymorphisms, our analysis focused on only three internationally studied SNPs. A more comprehensive genetic evaluation, including broader SNP panels within *ADIPOQ,* as well as variants in adiponectin receptor genes, may provide a more complete picture. Third, multiple comparisons were performed across variants and inheritance models without formal multiple-testing correction; thus, the findings should be regarded as exploratory and validated in independent cohorts. Finally, participants were recruited from a single hospital and may therefore not fully represent the metabolic and genetic diversity of the broader Vietnamese population.

## 4. Materials and Methods

Subject enrollment: Individuals were consecutively recruited at the University Medical Center between September 2023 and March 2024.

MtS was defined according to the 2005 IDF criteria [27]. Inclusion criteria for the MtS group were: (i) Vietnamese adults aged ≥18 years; (ii) increased WC (≥80 cm in females or ≥90 cm in males); and (iii) the presence of at least two of the following four MtS compo-nents: (1) elevated fasting glucose (≥5.6 mmol/L) or previously confirmed diabetes; (2) el-evated BP (systolic ≥ 130 mmHg or diastolic ≥ 85 mmHg) or previously confirmed hyper-tension; (3) hypertriglyceridemia (triglycerides ≥ 1.7 mmol/L); and (4) reduced HDL cho-lesterol (<1.29 mmol/L for females, <1.03 mmol/L for males). Participants were excluded from the MtS group if they had comorbidities that could affect metabolic parameters (e.g., uncontrolled hypothyroidism and advanced hepatic or renal disease).

For the control group, we recruited individuals without MtS and matched them to the MtS group by age and sex. Exclusion criteria for control participants were: (i) increased WC or (ii) confirmed diabetes mellitus. Individuals with increased WC were excluded because central obesity is the mandatory criterion in the IDF definition of MtS. Participants with confirmed diabetes mellitus were also excluded because diabetes has been shown to be associated with *ADIPOQ* polymorphisms in our previous study [26].

This observational study did not interfere with patients’ routine medical care and was conducted with full respect for participant comfort and confidentiality. The study protocol was approved by the Ethics Committee of the University of Medicine and Pharmacy at Ho Chi Minh City (protocol No 388/HĐĐĐ—ĐHYD, date of approval: 22 March 2023).

### 4.1. Assessment and Collection of Metabolic Parameters

Clinically, participants in both groups were initially screened based on the measurement of WC. They were then classified into the MtS or control group after verification of the remaining MtS components. Participants attending the hospital’s outpatient clinic who met the eligibility criteria for either the case or control group were invited to participate in the study. They were provided with detailed information regarding the study objectives and procedures, and informed consent was obtained prior to enrollment. Participants were also informed about the use of their blood samples for additional biochemical analyses, including the quantification of insulin and adiponectin levels, as well as the genotyping of selected *ADIPOQ* gene variants.

BP was measured in a seated position using an Omron automatic sphygmomanometer (Omron Healthcare, Kyoto, Japan) after the patient had rested for approximately 5–10 min. WC was measured by a plastic tape, following IDF guidelines, using a horizontal plane at the midpoint between the lower border of the last palpable rib and the iliac crest. Hip circumference was recorded at the level of the widest portion of the buttocks, with any obstructive clothing or items removed.

Fasting glucose and lipid profile parameters were assessed using Beckman Coulter assays (Beckman Coulter Co., Brea, CA, USA). Blood samples for insulin and adiponectin measurements were stored at −20 °C and transported to Hoa Hao Laboratory Center in the same city for analysis. Insulin concentrations were measured using a Roche Elecsys^®^ assay (Roche Diagnostics, Rotkreuz, Switzerland). Adiponectin levels were quantified using a Diazyme assay (Diazyme Laboratories, Poway, CA, USA). The assay principle relied on the aggregation of latex particles coated with anti-adiponectin antibodies, and the resulting increase in turbidity was quantified photometrically.

### 4.2. Genotyping

Blood samples used for deoxyribonucleic acid (DNA) extraction were collected in EDTA-containing tubes and transported to the Molecular Biomedical Center of the University of Medicine and Pharmacy for processing. DNA was isolated within 24 h following the GeneJET™ whole blood Genomic DNA Purification Kit protocol (Thermo Fisher Scientific, Waltham, MA, USA).

**Figure 3:**
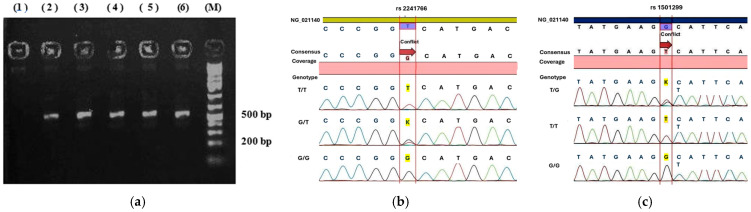
PCR amplification and Sanger sequencing of *ADIPOQ* polymorphisms rs2241766 and rs1501299. (**a**) Agarose gel electrophoresis of the 500 bp PCR amplicon encompassing exon 2 and intron 2 of the *ADIPOQ* gene. Lanes 2 to 6 represent DNA samples from patients; lane 1 is a negative control (H_2_O); lane M is the 1 kb Plus DNA ladder. (**b**) Representative Sanger sequencing chromatograms illustrating the three genotypes of rs2241766 (+45T>G): T/T, G/T, and G/G. (**c**) Representative chromatograms for rs1501299 (+276G>T), showing the G/G, T/G, and T/T genotypes. In panels (**b**) and (**c**), arrows indicate the polymorphic nucleotide position, the SNP site is marked by the vertical red lines, and the base call at the polymorphic position is highlighted in yellow. The letter “K” denotes the IUPAC ambiguity code for G/T. Abbreviations: SNP, single-nucleotide polymorphism; bp, base pair; IUPAC, International Union of Pure and Applied Chemistry.

Because rs2241766 (+45T>G) and rs1501299 (+276G>T) are located in close proximity within the *ADIPOQ* gene, a single primer pair was designed to amplify a DNA fragment encompassing both loci. The design was based on the human *ADIPOQ* reference sequence (NG_021140), obtained from the National Center for Biotechnology Information database, using CLC Main Workbench (Qiagen, Aarhus, Denmark). The forward primer was 5′GTAGACTCTGCTGAGATGGA3′, and the reverse primer was 5′GCACCACTACACTCATCCT3′, generating a 500 bp amplicon that covered both SNP sites (Figure 3a).

The fragment containing the rs2241766 and rs1501299 loci was amplified by polymerase chain reaction (PCR). The PCR products were verified on 2% agarose gels and enzymatically purified using ExoSAP–IT™ (Thermo Fisher Scientific, Waltham, MA, USA) prior to sequencing. Genotypes were determined by bidirectional Sanger sequencing using the BigDye Terminator v3.1 Cycle Sequencing Kit (Applied Biosystems, Waltham, MA, USA) on an ABI 3500 Genetic Analyzer. Alleles for rs2241766 (Figure 3b) and rs1501299 (Figure 3c) were identified by alignment with the *ADIPOQ* reference sequence.

**Figure 4:**
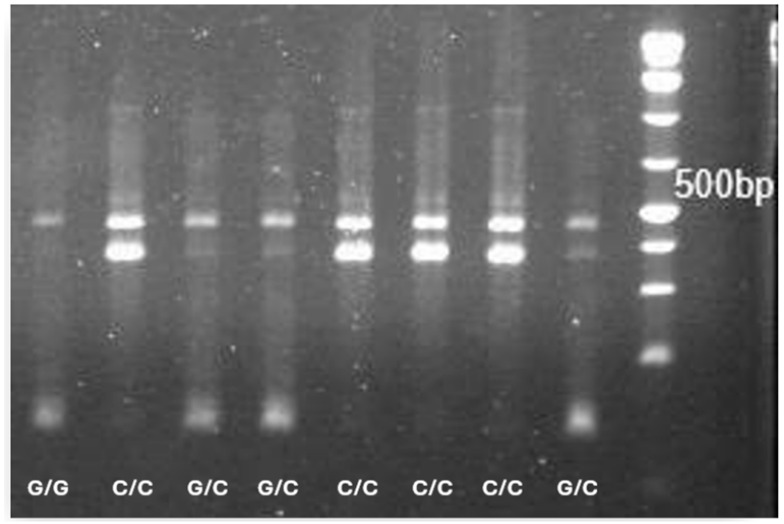
Electrophoretic patterns of rs266729 genotypes obtained by PCR. A control band of 447 bp is present in all lanes. The C/C genotype shows a specific band at 372 bp, the G/G genotype at 119 bp, and the G/C genotype displays both 372 bp and 119 bp bands. DNA ladder (rightmost lane) shows the 500 bp marker labeled.

For the rs266729 polymorphism, genotyping was carried out using a cost-effective refractory mutation system–PCR approach employing four specific primers: forward outer primer 5′TTGTTGAAGTTGGTGCTGGC3′, forward inner primer 5′CACGCTCATGTTTTGTTTTTGAGGC3′, reverse outer primer 5′GAACCGGCTCAGATCCTGCC3′, and reverse inner primer 5′GCTTGTGGCCTCGAATCGTA–3′. PCR products were separated on a 2% agarose gel to identify rs266729 genotypes (Figure 4). To ensure accuracy, twenty randomly selected samples were validated by Sanger sequencing.

### 4.3. Statistical Analysis

Distributional characteristics of continuous data were examined through histogram visualization and by assessing skewness and kurtosis values. Normally distributed data were expressed as mean ± standard deviation and were compared using Student’s *t*-test. In our dataset, non-normally distributed variables, including low-density lipoprotein, triglycerides, insulin, HOMA–IR, and adiponectin, are presented as median (IQR) and were compared using the Mann–Whitney U or Kruskal–Wallis, as appropriate. For categorical variables, group differences were analyzed using the chi-square test or Fisher’s exact test, as appropriate. All statistical analyses were performed using Stata 14.2 (StataCorp, College Station, TX, USA).

To explore the association between rs266729, rs2241766, and rs1501299 polymorphisms and MtS, analyses were performed using the web tool SNPStats. The associations were examined under multiple genetic inheritance models, including dominant, codominant, and log-additive models.

To compare adiponectin concentrations across genotypes, we calculated and compared the values for all three genotypes of each SNP in the total study population, as well as within the MtS and control groups. Additionally, we hypothesized that adiponectin levels would be lowest among individuals carrying the minor homozygous genotype of the studied SNPs; therefore, a non-parametric Cuzick trend test was performed to assess this association, and the corresponding *p* values for trends were reported.

To evaluate potential sex-specific genetic effects, we first conducted sex-stratified association analyses for each ADIPOQ variant across multiple inheritance models. Then, we performed SNP×sex interaction testing under the log-additive model; interaction *p* values were derived from the SNP×sex term, and sex-specific odds ratios were obtained from the interaction model.

## 5. Conclusions

Carriers of the G allele in rs266729 show an increased likelihood of MtS, whereas no significant associations were detected for rs2241766 or rs1501299. Nevertheless, adiponectin levels across all three variants tended to be lower or declined progressively, with an increasing number of minor alleles, particularly within the control group. These observations suggest that genetic variations in *ADIPOQ* may influence adiponectin levels at a very early stage, preceding the full clinical manifestation of MtS. Future investigations incorporating larger cohorts, broader genomic panels, and functional studies will be essential to fully clarify the biological pathways linking *ADIPOQ* variants to metabolic risk.

## Figures and Tables

**Table 1:** Comparison of anthropometric and metabolic parameters between controls and the MtS group.

Characteristics	Controls	MtS Group	*p*
Age (years)	47.61 ± 8.88	47.33 ± 9.07	0.784
Female	80 (50%)	80 (50%)	n/a
Weight (kg)	54.52 ± 6.72	69.74 ± 11.38	<0.001
Height (cm)	159.87 ± 7.92	161.69 ± 7.98	0.042
BMI (kg/m^2^)	21.32 ± 1.94	26.57 ± 3.0	<0.001
Waist circumference ^$^ (cm) Females Males	76.83 ± 5.66 74.35 ± 4.29 79.30 ± 5.79	92.91 ± 8.32 88.39 ± 6.13 97.43 ± 7.76	<0.001 <0.001 <0.001
Hip circumference ^$^ (cm) Females Males	89.83 ± 5.4 88.05 ± 4.68 91.59 ± 5.52	98.88 ± 6.29 96.83 ± 5.8 100.93 ± 6.12	<0.001 <0.001 <0.001
WHR ^$^ Females Males	0.86 ± 0.049 0.85 ± 0.046 0.87 ± 0.049	0.94 ± 0.07 0.91 ± 0.053 0.96 ± 0.075	<0.001 <0.001 <0.001
Increased WHR ^#^	52 (32.5%)	147 (91.88%)	<0.001
Systolic BP (mmHg)	120.94 ± 12.84	134.19 ±14.94	<0.001
Diastolic BP (mmHg)	77.12 ± 8.78	85.24 ± 11.35	<0.001
Fasting glucose (mmol/L)	5.09 ± 0.37	6.72 ± 1.56	<0.001
Triglycerides (mmol/L) *	1.18 (0.87–1.59)	2.41 (1.86–3.3)	<0.001
HDL cholesterol ^$^ (mmol/L) Females Males	1.38 ± 0.3 1.44 ± 0.26 1.32 ± 0.32	1.14 ± 0.25 1.20 ± 0.25 1.08 ± 0.23	<0.001 <0.001 <0.001
LDL cholesterol (mmol/L) *	3.36 (2.78–3.72)	3.48 (2.74–4.09)	0.189
Total cholesterol (mmol/L)	4.94 ± 0.81	5.26 ± 1.24	0.007
Fasting insulin (pmol/L) *	22.98 (16.14–35.46)	53.94 (34.32–81.6)	<0.001
Adiponectin (µg/mL) *	9.01 (6.09–12.45)	4.44 (2.39–6.83)	<0.001
HOMA–IR *	0.85 (0.61–1.32)	2.53 (1.65–3.98)	<0.001

Abbreviations: MtS, metabolic syndrome; BMI, body mass index; WHR, waist-to-hip ratio; BP, blood pressure; HDL, high-density lipoprotein; LDL, low-density lipoprotein; HOMA–IR, Homeostatic Model Assessment of Insulin Resistance; n/a, non–applicable. ^#^ WHR > 0.9 in males or WHR > 0.85 in females. * Variables are presented as median (interquartile range). ^$^ For these variables, sex-stratified values are shown below the overall estimates, and *p* values compare MtS versus controls within each sex.

**Table 2:** Distribution of MtS components in the control and MtS groups.

MtS Components *	Controls	MtS Group	*p*
Increased waist circumference	0	160 (100%)	n/a
Elevated fasting blood glucose	11 (6.88%)	134 (83.75%)	<0.001
Elevated BP	46 (28.75%)	130 (81.25%)	<0.001
Decreased HDL cholesterol	31 (19.38%)	88 (55%)	<0.001
Elevated triglycerides	26 (16.25%)	127 (79.38%)	<0.001
Number of MtS components:			
0	73 (45.6%)	0	<0.001
1	60 (37.5%)	0	
2	27 (16.9%)	0	
3	0	40 (25)	
4	0	81 (50.6%)	
5	0	39 (24.4%)	

Abbreviations: MtS, metabolic syndrome; BP, blood pressure; HDL, high-density lipoprotein; n/a, non–applicable. * MtS components are defined according to 2005 International Diabetes Federation guidance.

**Table 3:** Genotypic distribution of *ADIPOQ* variants (rs266729, rs2241766, rs1501299) and their relations with MtS across various inheritance models.

SNPs	Genotypes	Control Group	MtS Group	OR (95% CI)	*p*
rs266729 (C>G)					
Codominant	C/C	107 (66.9%)	88 (55%)	1	0.092
	G/C	44 (27.5%)	59 (36.9%)	1.63 (1.01–2.64)	
	G/G	9 (5.6%)	13 (8.1%)	1.76 (0.72–4.30)	
Dominant	C/C	107 (66.9%)	88 (55%)	1	0.029
	G/C—G/G	53 (33.1%)	72 (45%)	1.65 (1.05–2.60)	
Recessive	C/C—G/C	151 (94.4%)	147 (91.9%)	1	0.38
	G/G	9 (5.6%)	13 (8.1%)	1.48 (0.62–3.58)	
Log-additive				1.46 (1.02–2.09)	0.038
rs2241766 (T>G)					
Codominant	T/T	61 (38.1%)	70 (43.8%)	1	0.59
	G/T	71 (44.4%)	64 (40%)	0.79 (0.49–1.27)	
	G/G	28 (17.5%)	26 (16.2%)	0.81 (0.43–1.53)	
Dominant	T/T	61 (38.1%)	70 (43.8%)	1	0.31
	G/T—G/G	99 (61.9%)	90 (56.2%)	0.79 (0.51–1.24)	
Recessive	T/T—G/T	132 (82.5%)	134 (83.8%)	1	0.77
	G/G	28 (17.5%)	26 (16.2%)	0.91 (0.51–1.64)	
Log-additive				0.88 (0.65–1.19)	0.39
rs1501299 (G>T)					
Codominant	G/G	90 (56.2%)	90 (56.2%)	1	0.64
	G/T	57 (35.6%)	65 (40.6%)	1.25 (0.79–1.99)	
	T/T	13 (8.1%)	13 (8.1%)	1.10 (0.48–2.50)	
Dominant	G/G	90 (56.2%)	82 (51.2%)	1	0.37
	G/T—T/T	70 (43.8%)	78 (48.8%)	1.22 (0.79–1.90)	
Recessive	G/G—G/T	147 (91.9%)	147 (91.9%)	1	n/a
	T/T	13 (8.1%)	13 (8.1%)	1.00 (0.45–2.23)	
Log-additive				1.13 (0.80–1.59)	0.49

Abbreviations: MtS, Metabolic syndrome; SNP, single-nucleotide polymorphism; OR, odds ratio; CI, confidence intervals; n/a: non-applicable.

**Table 4:** Median adiponectin concentrations (interquartile ranges) in relation to genotypes of rs266729, rs2241766, and rs1501299 under comparison and trend tests.

SNPs	Genotypes			*p* for Comparison ^a^	*p* for Trend ^b^
rs266729	C/C	G/C	G/G		
Total population	6.69 (3.84–10.52)	6.55 (3.32–9.52)	4.28 (3.14–6.38)	0.038	0.036
Control group	9.35 (6.31–13.07)	9.18 (6.25–11.75)	5.59 (3.97–7.21)	0.028	0.075
MtS group	4.24 (2.47–6.74)	5.39 (2.06–7.13)	4.08 (2.32–5.57)	0.680	0.838
rs2241766	T/T	G/T	G/G		
Total population	6.59 (2.85–12.34)	6.88 (4.26–10.69)	6.09 (3.25–8.41)	0.044	0.083
Control group	11.84 (3.95–17.21)	9.35 (6.88–12.49)	7.21 (5.56–10.62)	0.052	0.020
MtS group	3.58 (2.15–7.83)	4.93 (3.08–6.80)	4.89 (1.98–6.90)	0.386	0.074
rs1501299	G/G	T/G	T/T		
Total population	6.81 (3.49–10.18)	6.94 (6.31–10.17)	4.45 (3.16–7.75)	0.150	0.081
Control group	9.35 (6.58–14.79)	8.76 (5.94–11.75)	5.56 (3.55–9.06)	0.095	0.044
MtS group	4.15 (2.34–7)	5.16 (2.61–6.53)	3.84 (1.47–7.02)	0.656	0.777

Abbreviations: Mts, metabolic syndrome; SNP, single-nucleotide polymorphism. ^a^ Kruskal–Wallis test, ^b^ Cuzick’s non-parametric test for trend.

## Data Availability

Data supporting the findings of this study are available from the corresponding author upon reasonable request.

## Supplementary Materials

The following supporting information can be downloaded at: https://www.mdpi.com/article/10.3390/ijms27041780/s1.

## Abbreviations

The following abbreviations are used in this manuscript:

MtS	Metabolic syndrome
BP	Blood pressure
SNP	Single-nucleotide polymorphism
WC	Waist Circumference
HDL	High-density lipoprotein
IDF	International Diabetes Federation
IQR	Interquartile Range
PCR	Polymerase chain reaction
DNA	Deoxyribonucleic acid
LD	Linkage disequilibrium
HWE	Hardy–Weinberg equilibrium
