# Supplementary material for: An Exploratory Study of ADIPOQ Polymorphisms, Adiponectin Levels and Metabolic Syndrome in a Vietnamese Population
Source: Int J Mol Sci. 2026 Feb 12;27(4):1780. doi: 10.3390/ijms27041780 (PMC12941168; doi:10.3390/ijms27041780)
Supplement: Supplementary file 1 [file ijms-27-01780-s001.zip › ijms-4106729-supplementary.pdf]

Table S1. Multivariable linear regression of log-transformed adiponectin concentrations.

Predictor	β	95% CI	p
Male (vs female)	-0.424	-0.567 to -0.280	<0.001
Age (per 1-year increase)	0.023	0.015 to 0.031	<0.001
MtS group (vs control)	-0.76	-0.903 to -0.617	<0.001

Abbreviations: MtS, metabolic syndrome; β, regression coefficient; CI, confidence intervals.

Table S2. Genotype distributions of ADIPOQ variants (rs266729, rs2241766, rs1501299) in females and their associations with MtS under different genetic models.

SNPs	Genotypes	Control group	MtS group	OR (95% CI)	p
rs266729 (C>G)					
Codominant	C/C	56 (70.0%)	46 (57.5%)	1	0.17
	G/C	19 (23.8%)	30 (37.5%)	1.92 (0.96–3.85)	
	G/G	5 (6.2%)	4 (5%)	0.97 (0.25–3.84)	
Dominant	C/C	56 (70%)	46 (57.5%)	1	0.099
	G/C – G/G	24 (30%)	34 (42.5%)	1.72 (0.90–3.31)	
Recessive	C/C – G/C	75 (93.8%)	76 (95%)	1	0.73
	G/G	5 (6.2%)	4 (5%)	0.79 (0.20–3.05)	
Log-additive				1.38 (0.81–2.33)	0.23
rs2241766 (T>G)					
Codominant	T/T	31 (38.8%)	33 (41.2%)	1	0.91
	G/T	32 (40%)	32 (40%)	0.94 (0.47–1.88)	
	G/G	17 (21.2%)	15 (18.8%)	0.83 (0.35–1.94)	
Dominant	T/T	31 (38.8%)	33 (41.2%)	1	0.31
	G/T – G/G	49 (61.2%)	47 (58.8%)	0.90 (0.48–1.70)	
Recessive	T/T – G/T	63 (78.8%)	65 (81.2%)	1	0.69
	G/G	17 (21.2%)	15 (18.8%)	0.86 (0.39–1.86)	
Log-additive				0.91 (0.60–1.38)	0.67
rs1501299 (G>T)					
Codominant	G/G	48 (60%)	45 (56.2%)	1	0.67
	G/T	27 (33.8%)	27 (33.8%)	1.07 (0.55–2.09)	
	T/T	5 (6.2%)	8 (10%)	1.71 (0.52–5.60)	
Dominant	G/G	48 (60%)	45 (56.2%)	1	0.63
	G/T – T/T	32 (40%)	35 (43.8%)	1.17 (0.62–2.19)	
Recessive	G/G – G/T	75 (93.8%)	72 (90%)	1	0.38
	T/T	5 (6.2%)	8 (10%)	1.67 (0.52–5.33)	
Log–additive				1.20 (0.74–1.96)	0.46

Abbreviations: MtS, metabolic syndrome; SNP, single-nucleotide polymorphism; OR, odds ratio; CI, confidence intervals.

Table S3. Genotype distributions of ADIPOQ variants (rs266729, rs2241766, rs1501299) in males and their associations with MtS under different genetic models.

SNPs	Genotypes	Control group	MtS group	OR (95% CI)	p
rs266729 (C>G)					
Codominant	C/C	51 (63.8%)	42 (52.5%)	1	0.210
	G/C	25 (31.2%)	29 (36.2%)	1.41 (0.72–2.76)	
	G/G	4 (5%)	9 (11.2%)	2.73 (0.79–9.50)	
Dominant	C/C	51 (63.8%)	42 (52.5%)	1	0.150
	G/C – G/G	29 (36.2%)	38 (47.5%)	1.59 (0.85–3.00)	
Recessive	C/C – G/C	76 (95%)	71 (88.8%)	1	0.140
	G/G	4 (5%)	9 (11.2%)	2.41 (0.71–8.17)	
Log-additive				1.54 (0.94–2.53)	0.084
rs2241766 (T>G)					
Codominant	T/T	30 (37.5%)	37 (46.2%)	1	0.490
	G/T	39 (48.8%)	32 (40%)	0.67 (0.34–1.30)	
	G/G	11 (13.8%)	11 (13.8%)	0.81 (0.31–2.13)	
Dominant	T/T	30 (37.5%)	37 (46.2%)	1	0.260
	G/T – G/G	50 (62.5%)	43 (53.8%)	0.70 (0.37–1.31)	
Recessive	T/T – G/T	69 (86.2%)	69 (86.2%)	1	n/a
	G/G	11 (13.8%)	11 (13.8%)	1.00 (0.41–2.46)	
Log-additive				0.83 (0.53–1.31)	0.420
rs1501299 (G>T)					
Codominant	G/G	41 (51.2%)	37 (46.2%)	1	0.450
	G/T	31 (38.8%)	38 (47.5%)	1.36 (0.71–2.60)	
	T/T	8 (10%)	5 (6.2%)	0.69 (0.21–2.31)	
Dominant	G/G	41 (51.2%)	37 (46.2%)	1	0.530
	G/T – T/T	39 (48.8%)	43 (53.8%)	1.22 (0.66–2.27)	
Recessive	G/G – G/T	72 (90%)	75 (93.8%)	1	0.380
	T/T	8 (10%)	5 (6.2%)	0.60 (0.19–1.92)	
Log–additive				1.20 (0.74–1.96)	0.460

Abbreviations: MtS, metabolic syndrome; SNP, single–nucleotide polymorphism; OR, odds ratio; CI, confidence intervals; n/a: non–applicable.

Table S4. SNP×sex interaction analysis for MtS under the log-additive model.

SNPs	Sex	OR (95% CI)	p	p for interaction
rs266729 (C>G)	Females	1.37 (0.81–2.33)	0.235	0.760
	Males	1.54 (0.94–2.52)	0.088	
rs2241766 (T>G)	Females	0.91 (0.60–1.38)	0.657	0.762
	Males	0.83 (0.53–1.31)	0.422	
rs1501299 (G>T)	Females	1.21 (0.74–1.95)	0.441	0.666
	Males	1.03 (0.63–1.68)	0.892	

Abbreviations: MtS, metabolic syndrome; SNP, single-nucleotide polymorphism; OR, odds ratio; CI, confidence intervals. Sex-specific ORs were obtained from interaction models; p for interaction corresponds to the SNP×sex term.
